# Utilizing SEM-RFC to predict factors affecting online shopping cart abandonment during the COVID-19 pandemic

**DOI:** 10.1016/j.heliyon.2022.e11293

**Published:** 2022-10-28

**Authors:** Ardvin Kester S. Ong, Marjorie Joy R. Dejucos, Mary Anne F. Rivera, John Vincent D.J. Muñoz, Miguel S. Obed, Kirstien Paola E. Robas

**Affiliations:** a School of Industrial Engineering and Engineering Management, Mapúa University, 658 Muralla St., Intramuros, Manila 1002, Philippines; b Department of Industrial Engineering, Faculty of Engineering, University of Santo Tomas, España Blvd, Manila 1015, Philippines

**Keywords:** Online shopping, Random forest classifier, Structural equation modeling, Shopping cart abandonment

## Abstract

Online shopping has accelerated during to the pandemic and an increase in online shopping cart abandonment (SCA) was also evident. The growth of online shopping is contributed by the rising middle class, high consumer spending, millennials, and a tech-savvy population which is valuable to the growth of e-commerce. This study aimed to predict the factors that affect SCA during the COVID-19 Pandemic utilizing the SEM-RFC hybrid. Several factors such as self-efficacy, attribute conflicts, hesitation at checkout, emotional ambivalence, choice process satisfaction, attitude, subjective norms, and perceived behavioral control were analyzed simultaneously. This study integrated the cognition-affect-behavior paradigm with the Theory of Planned Behavior to provide a conceptual framework measured through an online survey questionnaire answered by 1015 valid responses collected by convenience sampling. Results showed that Attitude, Attribute Conflict, Self-Efficacy, and Emotional Ambivalence are the primary significant factors affecting SCA. Amidst the pandemic, consumers still value the ease of use, convenience and safety of the mobile online shopping applications that they have, which they do not positively experience at this time. The findings of this study may be applied and extended by researchers, online retailers, and businesses to understand consumer's abandonment intentions. Moreover, the results and framework of this study may be capitalized on by the business sector to create marketing strategies and develop business models for a sustainable online shopping business worldwide.

## 1 Introduction

E-commerce has evolved as merchandisers continue to innovate in order to reach customers, especially during the COVID-19 pandemic. The website, Multiply, started to bring e-commerce into the mainstream scene due to its popularity in the early 2000s. Multiply became a huge social media site among Filipinos which then garnered more than 90 thousand online sellers by 2011 (Reyes, 2016). It was eventually known as “Multiply Shopping” and became a social shopping site for entrepreneurs to sell a wide range of products and services. During that time, the Philippines was said to be the largest and fastest growing e-market for Multiply (Ho, 2011). This growth continued to expand by 2012 when it was reported that the site garnered approximately 5.5 million users in the Philippines, 130 thousand of which are online store fronts carrying items from 16 product categories (Periabras, 2012).

**Figure 1:**
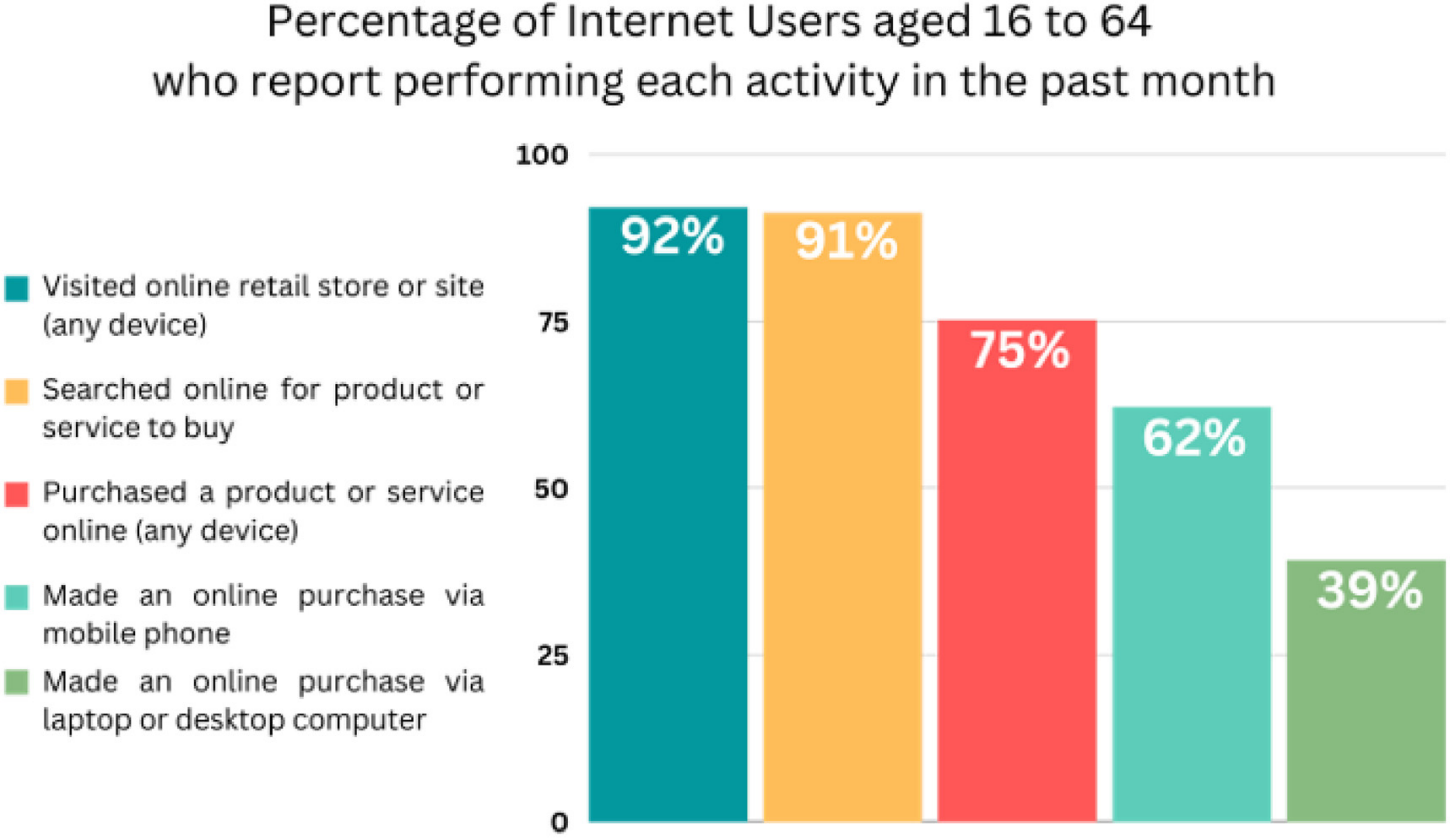
E-commerce activities (Jiang et al., 2021; Data Reportal, 2021).

In the present, e-commerce has grown parallel to the rise of technology as more people started to use the digital platforms as a market. According to Katrina B. and Benedict L. (2021), there are 67% users in the 107.3 million population and 36.23% were able to purchase product and services online. From the population who are active online shoppers, the demographics covers ages between 16-64 years old. These online shopping activities include searching for products or services to buy, visiting online retail stores or sites, purchasing a product or service, and having made an online purchase through various devices such as laptops, desktop computers, or mobile phones. Figure 1 presents the breakdown of e-commerce activities (Jiang et al., 2021).

The COVID-19 pandemic accelerated e-commerce adoption, which is likely to continue in the coming months with lockdown measures and the new variants of the COVID-19 virus. The reason for the adoption in e-commerce is due to the safety measures against the COVID-19 pandemic (Ong et al., 2021). During the lockdown period of 2020, shoppers increased their basket size by 57% compared to the previous years, Philippines as one of the biggest growths in Southeast Asia (Nonato, 2020). From the report of Magkilat (2020), one of the highest usages with an increase with Android phones is the Philippines. It was seen that 53% of the second quarter in 2020 was significantly higher than the first quarter. Around 4.9 billion total session for online shopping applications were seen. In addition, the e-commerce such as Lazada and Shoppe (a multinational company) rose and opted to change their strategies during the pandemic to adapt with the current situation. It was also seen that e-commerce had the main responsibility to create business models and strategies with the opportunities available in the online market (Magkilat, 2020). This responsibility accompanies the problem of online shopping cart abandonment (SCA) that continues to be a widespread challenge across e-commerce sites.

**Figure 2:**
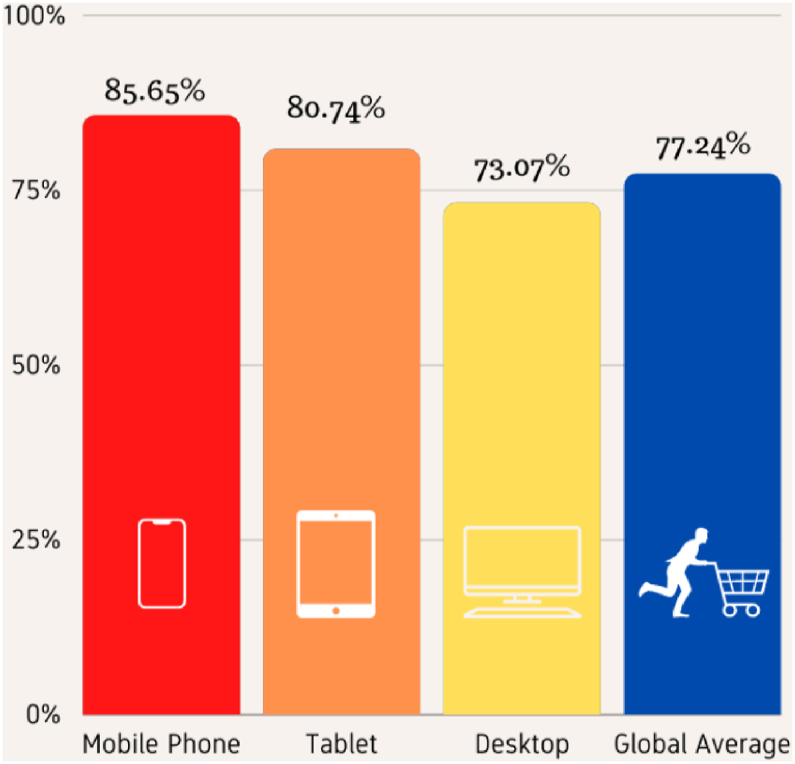
Cart abandonment rate statistics by device.

SCA is when a user adds an item to an e-commerce site's online shopping cart but does not go to checkout and finish the transaction. The shopping orders abandonment rate worldwide is 88.5% (Statista, 2021a, Statista, 2021b, Statista, 2021c, Statista, 2021d). As seen in Figure 2, abandoned shopping carts are correlated to the different devices being utilized by consumers. From the 2015 report on cart abandonment rate statistics by device, 85.65% of all transactions end without a sale, and mobile phones have the highest cart abandonment rates. Tablets, on the other hand, are converted sales 80.74% of the time, resulting in a 5.7% increase in revenue. Cart abandonment rates were highest on desktops, with 73.07% of transactions failing (Serrano, 2022).

The firm loses money when potential consumers choose the exit button rather than the “complete your order” button. According to Forrester's research, SCA costs e-commerce businesses an estimated $18 billion in annual sales income (Fox, 2020). Some of the reasons why people abandon their shopping carts are (1) just looking (34%), (2) Shipping cost and options (23%), (3) comparing prices (18%), (4) to buy in-store (15%), (5) lack of payment options (6%), and (6) technical issues (4%) (Sale Cycle Abandonment Surveys, 2021). According to Toth (2019), while 100 customers put something into the cart, only 20 finish a checkout transaction. This means that the cart abandonment rate is 80% (e.g., the business loses $400 on every $100 of revenue).

Worldwide, online SCA rate has been ranging from 59.8% to 71.98% from 2006 to 2020. All regions have an average of 74.52% cart abandonment rate which represents the percentage of customers who left their carts behind instead of purchasing. In another survey conducted by iVend Retail in 2018, online SCA continues to be a challenge in e-commerce. To which, 34.5% of consumers stated they only use the shopping cart as a wish list. This number is even higher in the Philippines at 41.6%, followed by Mexico at 41.2%. Meanwhile, Canada and Germany tied at 40.4%. With the information gathered, Peterson (2021) stated that there are more huge impacts of the SCA beyond the loss of revenue.

It was seen that consumers who are abandoning their SCA are worth higher money than that they spent on their first transaction. Product inventory and availability issues arise from SCA. Reserved items in the carts could not be sold to other consumers and product unavailability will result in loss of sales because real customers who are ready to buy cannot purchase the items. Abandoned carts have a big influence on the analytics dashboard's statistics resulting in creating business strategies based on inaccurate data. Unsavable abandoned carts can mess with a business' advertising audiences because every time someone adds an item to their cart, history and cookies are created, which might cause inaccurate advertising targets. In extreme cases, cart abandonment could cause slower site performance. Therefore, the need to analyze SCA among consumers should be explored since it is seen that consumers are likely to have the behavior. To measure the behavior of consumers, theories such as the theory of planned behavior (TPB) (Ong et al., 2021) and cognition-affect-behavior paradigm (CAB) may be utilized (Huang et al., 2018).

Cho (2003) discovered components that led to the abandonment of shopping carts, such as a lack of physical scrutiny, worries about delivery and return, frequent catalog purchases, and previous online purchasing experiences. Risk perceptions were shown to have a direct detrimental impact on abandonment. Kumar et al. (2018) found that online buying was highly connected with information search and convenience. According to Egeln and Joseph (2012), the strength of perceived ownership in intended activity and perceived ownership in actual behavior have a significant positive link. According to Moore and Mathews (2006), the most considerable risk connected with purchasing online was performance and the financial risks linked with the transaction itself. A major research conducted by Baymard Institute showed 68.81% of shopping carts are abandoned on the internet. To which, a three times loss on the profit among online retailers were evident, reaching to approximately $18 million every year. The challenge in the navigation of web pages, unexpected costs with products, competition having better prices, and long processes with excessive difficulties in security payments, and an inadequate delivery choice were the top causes for cart abandonment. Despite the fact that there are several reasons for SCA that are unrelated to reaction time, this study is considered underexplored relating to the factors affecting consumer behavior. These led to different research questions where the objective of this study was built upon.

1. What are the significant factors affecting consumer's behavioral intention for shopping cart abandonment?
2. Can the integrated TPB and CAB holistically measure the behavioral intentions of consumer?
3. Was the results consisted in the analysis conducted to justify the findings?
4. What theoretical and practical implications can be drawn?
5. What managerial implications would be created based from the findings of the study?

The objective of this study was to predict factors affecting online SCA among consumers during the COVID-19 pandemic. This study identified the significant variables that positively affect consumers' behavior and decision processes related to the problem. Many e-commerce businesses have the problem of not having an abandonment reduction plan in place or not knowing where to start when developing one. Understanding these factors give in-depth knowledge about consumers’ abandonment intentions that is useful to create business strategies. With the integration of CAB and TPB, this study simultaneously analyzed several factors such as attitude, perceived behavioral control, and subjective norm under TPB. In addition, factors such as attribute conflict, self-efficacy, emotional ambivalence, hesitation to check-out, and choice-process satisfaction under CAB were considered to determine factors affecting shopping cart abandonment. Structural equation modeling (SEM) with random forest classifier (RFC) hybrid was utilized in this study following the suggestion of Duarte and Pinho (2019).

Duarte and Pinho (2019) stated how SEM may utilize another tool to verify its findings. Woody (2011) stated how the mediators in SEM may cause a lower significance level since the independent variables are influenced. In addition, Fan et al. (2016) explained how the father the independent variable to the dependent variable may cause low to no significance level. Thus, RFC as a machine learning tool has been considered for the classification of significant factors hybrid to the SEM result. The results of this study may be a way to help retailers understand how consumers chose to invest their available assets like money, effort, and time while buying products. In addition, the retailers of various online shops take advantage of this research in terms of understanding and taking into consideration the consumer's behavior in the market. This study is the first study that completely analyzed factors affecting consumer's online shopping cart abandonment behavior during the COVID-19 pandemic. Lastly, the results and framework of this study may be applied and extended among other online businesses, even in-store businesses worldwide.

## 2 Conceptual framework

Several studies in the present have conducted analysis in the field of online SCA. Song (2019) dealt heavily on different motivational factors affecting abandonment. Rubin et al. (2020) considered abstract and concrete mindsets among consumers. Moore and Mathews (2006) considered brands, design, reputation of brands, and price on the overall performance risk and extrinsic cues for online SCA. On the other hand, Wang et al. (2022) focused on stimulus-organism-response model. Hesitation at checkout promoted the online SCA based from their findings. Jiang et al. (2021) focused on the post-decision stage among consumers to assess online SCA. Lastly, Mishra et al. (2021) considered moderating effects of SCA and value-consciousness. The CAB paradigm when it comes to online SCA was considered by Huang et al. (2018). It was seen from their study that significant factors under CAB were considered important constructs. However, their limitations presented the inconsideration of motivational such as behavioral variables. Their suggestion was to examine several factors affecting SCA through integration or extension of the CAB paradigm.

In accordance to the different recent studies, there has been a lack of studies that considered the holistic measurement of check-out behavioral problems. Based from the different studies, it was deduced that the CAB model considered by Huang et al. (2018) measured the motivation and emotional aspect of online SCA completely. However, as suggested, the behavioral aspects should also be explored to analyze completely SCA. With that, TPB was considered as a framework that has been denounced as a model which completely analyze an individual's behavior (German et al., 2022). Prasetyo et al. (2020) also utilized the framework in assessing online food delivery among consumers in the Philippines. Similarly, Gumasing et al. (2022) considered the TPB to analyze intention to use online grocery. Lastly, Ong et al. (2021) assessed clothing apparel online shopping. It was seen that TPB extended or integrated with other models would completely cover the behavioral aspect of an individual; through which, the integration with CAB would cover the emotional and cognitive aspect of an individual in online SCA.

**Figure 3:**
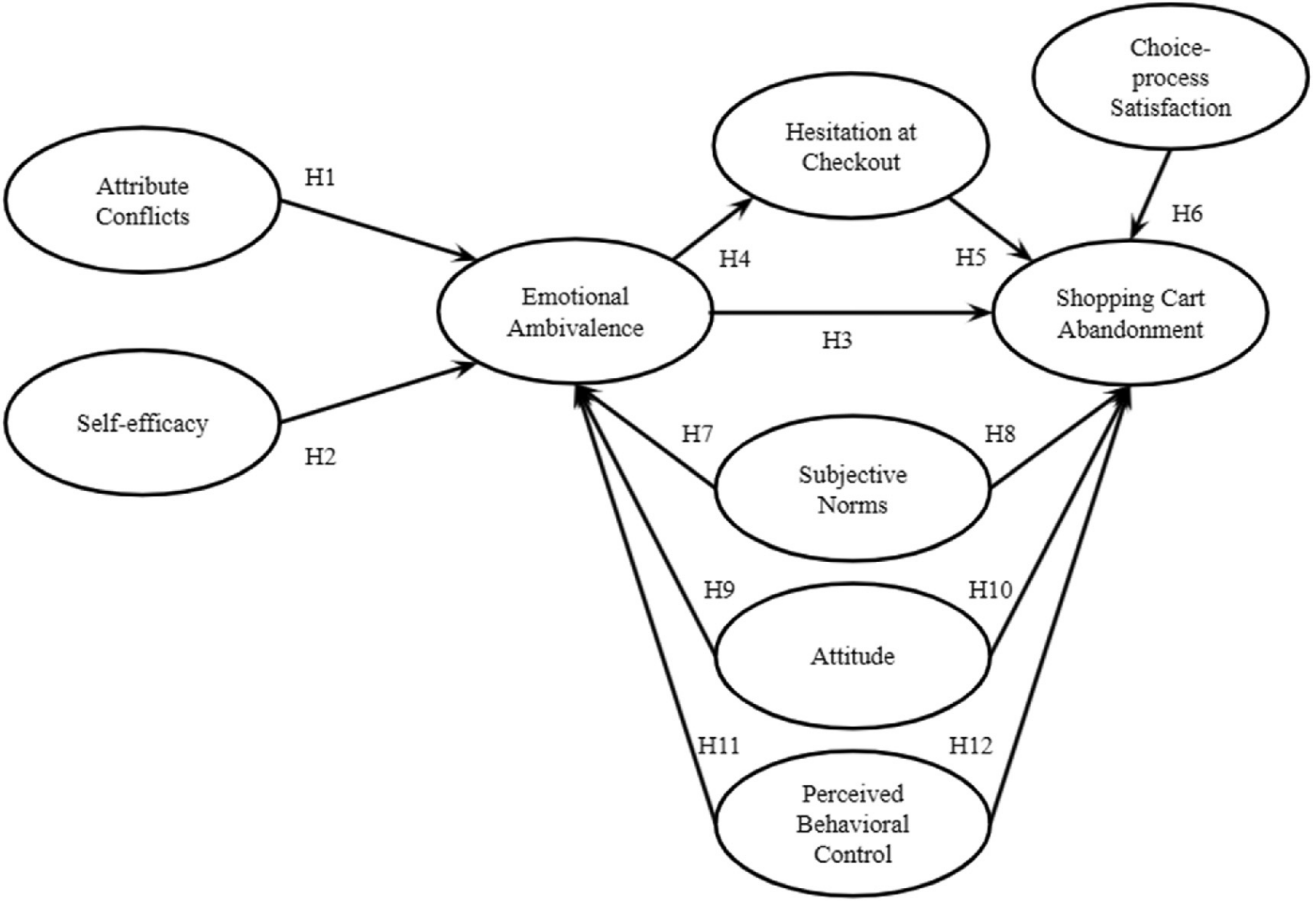
Conceptual framework.

The current research fills the gap by extending the existing conceptual framework of CAB (Huang et al., 2018) by examining the role of the TPB (Ong et al., 2021) within online shopping cart abandonment. Twelve (12) hypotheses were developed and considered in this study. A total of 9 latent variables were analyzed simultaneously using SEM and RFC hybrid. Presented in Figure 3 is the conceptual framework utilized in this study. Following which is the discussion of related studies and hypotheses development.

The framework of the study shows the cognition-affect-behavior paradigm (CAB) in which Huang et al. (2018) described as the representation of the formation process of behavior. It was seen from the created framework that the thoughts on the affective reaction may be considered unfavorable (favorable) which is an antecedent of behavioral intentions. The framework was utilized as a basis to characterize and explain consumer behavior. It also involves factors that are hypothesized to have significant relationships with one another that may lead to online SCA.

Intrapersonal conflicts or attribute conflict may be considered as the positive (negative) reaction by an individual, while interpersonal conflict is the agreement (disagreement) by an individual with another person. This objective assessment is present as an antecedent of affected-oriented ambivalence. To which, self-efficacy is proposed under intrapersonal conflicts which covers the ability of an individual to carry out a decision regarding a goal.

Ambivalence is described in this study as the incompatibility of cognitions or emotions. Emotional ambivalence (EA) as defined by Huang et al. (2018) is the evasive feelings when purchasing which may be caused by cognition or emotions. Moreover, the hesitation at check-out is an antecedent that affects EA and SCA as presented in the study of Huang et al. (2018). As an extension to the framework, constructs from the TPB are proposed to have a relationship with SCA. This may represent a customer's actual control over their behavior. The concept of TPB anticipates a person's behavior, leading to intention for a certain behavior (Ong et al., 2021).

### 2.1 Hypotheses development

The pandemic caused an increase in online shopping in the Philippines. The expanding middle class, increased consumer spending, millennials, and a tech-savvy populace are all factors that contribute to the expansion of online shopping. The occurrence of online SCA, on the other hand, is one of the primary concerns hurting the e-commerce business (Sondhi, 2017). To which, attitudinal inconsistency in one form of psychological instability that led to different mixed emotions. Uncomfortable tensions result when cognitive dissonance occurs (Lee and Aaker, 2004). Consumers who have highly positive and highly negative views tend to have more ambivalent emotions (Russell & Klein, 2011). This then affects self-efficacy (SE) since it is also inclined with aversion and discomfort. SE in this study is determined through the perception of an individual with their capability to handle their emotions (Fullagar et al., 2013). Discomfort can lead to negative effects on satisfaction (Chea and Luo, 2008). Therefore, it was hypothesized that:

H1 Attribute conflicts (AC) have a direct significant effect on emotional ambivalence (EA).

H2 Self-efficacy (SE) has a direct significant effect on emotional ambivalence (EA).

Negative emotions could dominate an individuals' decision-making process. Getting away from the thing or incident is one way to prevent negative emotions. Discomfort and aversion are also linked to EA (van Harreveld et al., 2009). Affliction has been demonstrated to negatively impact brand perceptions (Chang, 2011) and satisfaction (Chea and Luo, 2008). It could be said that EA thus raises the chance of SCA. Therefore, it was hypothesized that:

H3 Emotional ambivalence (EA) has a significant direct effect on shopping cart abandonment (SCA).

The postponement of purchasing due to processing time needed for final decision making is considered as online shopping hesitation or hesitation at check-out (HC). Basically, HC, SCA, and overall hesitation are the three types and implies that consumers’ ability is limited only in the early stage of purchase when it comes to online shopping. Cho et al. (2006) indicated that the risk due to mobile shopping creates the EA in proceeding to the checkout stage. In addition, for smartphone users, consumers are more hesitant when they are more ambivalent. Thus, it was hypothesized that:

H4 Emotional ambivalence (EA) has a direct effect on hesitation at check-out (HC).

Hesitation at check-out is defined as a person's ability to be undecided for purchasing an item once considered. Researchers have been trying to understand why nearly two-thirds of customers abandon their online shopping carts. This phenomenon is also known as SCA. The process involves the consumer's search and selection of the product and cancellation of the intended purchase occurs towards checkout (Lee, B. & Lee W., 2004). To which, those who purchase with more hesitation are more inclined to abandon their carts. Thus, it was hypothesized that:

H5 Hesitation at check-out (HC) has a direct effect on shopping cart abandonment (SCA).

Choice-process satisfaction is defined as the option of an individual to choose among all equally available options in accordance to their liking. Ekta et al. (2020) studied how consumers are affected with cashless transactions. Results showed that consumers are happy and satisfied with cashless payment solutions. The significant advantage of cashless transactions, in the view of the consumer, is the enhanced security. Data suggest that consumer satisfaction and product quality are linked. The variables have a positive impact on the customer's overall satisfaction. Moreover, Huang et al. (2018) stated that the hesitation at cart abandonment relationship is moderated by choice-process satisfaction (SAT), and the link is weaker at greater levels of SAT. With the option of payment method, this choice-process may affect a consumer's satisfaction and may reduce the risk of SCA. Thus, it was hypothesized that:

H6 Choice-process satisfaction (SAT) has a direct significant effect towards shopping cart abandonment (SCA).

Subjective norm as defined under TPB is the influence of people around an individual towards their behavior (Ong et al., 2021b). Priester (2002) explained how stress may be preceded by EA and subjective norm (SN) may be a feasible approach of decreasing ambivalence, and individuals turn to others for advice on how to behave in the absence of tangible knowledge (Suls and Wheeler 2000). According to this logic, adolescents with EA should experience increased evaluative strain, which should push them to minimize emotion decisiveness by acting on the SN of key collaboration in directing their thoughts and acts. Thus, it was hypothesized that:

H7 Subjective norms (SN) has a direct significant effect towards emotional ambivalence (EA).

According to Hsu et al. (2006), when faced with a decision on how to act, consumers consider how significant others act or think one should act. As a result, there appears to be a positive link between SN and behavioral intentions. In practical terms, this indicates that positive opinions will drive consumers to buy things on the internet, while bad evaluations will have the opposite effect. In addition, SN was indicated to have the most significant effect when purchasing a product online (Ong et al., 2021). Therefore, it was hypothesized that:

H8 Subjective norms (SN) has a direct significant effect towards shopping cart abandonment (SCA).

Attitude is a person's feeling and evaluation of the behavior (Ong et al., 2021). According to the study of Priester (2002), EA are the cognition and emotional aspect that leads to confliction either positive or negative, a feature of an individual's attitude (A). To which, this conflict creates an aversive appraisal where the individual may not be aware of. If one of the components of Attitude (A) were to change, bringing it into congruence with the other components, the ambivalence would reduce (Huang et al., 2018). This could lead to the conscious decision-making of one individual (Ong et al., 2021). Therefore, it was hypothesized that:

H9 Attitude (A) has a direct significant effect towards emotional ambivalence (EA).

Attitudes are defined as “the degree to which an individual's feelings toward a psychological object are favorable or unfavorable” (Ajzen and Fishbein, 2000). They have a substantial predictive capacity on intentions to do a specific behavior, with more positive attitude being associated with higher behavioral intentions. Perceived behavioral control on the other hand, is an individual's control and belief that they have the autonomy to decide over a specific action (Ajzen and Fishbein, 2000; Ong et al., 2021). Studies regarding shopping online have confirmed the relationship (Verhoef and Langerak, 2001; Ong et al., 2021; Khatibi et al., 2006; Haque and Khatibi, 2005; Suki et al., 2008) even in online shopping rate (Haque and Khatibi, 2005; Wu, 2003; Yang et al., 2007; Khatibi et al., 2006). Greater levels of A are associated with significantly higher purchase intentions. In addition, moderating the role of EA within the TPB, A, SN, perceived behavioral control (PBC), behavioral intentions, and self-reported behavior was seen to be evident (Conner et al., 2003). It shows that the attitude-behavior and PBC relationship are weaker in higher ambivalence than of lower ambivalence. Thus, it was hypothesized that:

H10 Attitude (A) has a direct significant effect on shopping cart abandonment (SCA).

H11 Perceived behavioral control (PBC) has a direct significant effect towards emotional ambivalence (EA).

Trust is one of the PBC that determine abandoning behavior (George, 2004; Hsu et al., 2011). However, according to Egeln and Joseph (2012), SCA occurs after the purchase decision has been made. As a result, TPB fails to account for the moderating influence of any circumstance that causes a shopping cart to be abandoned after a purchase decision has been made. However, other studies (Ong et al., 2021) presented how PBC can positively affect the decision of an individual upon purchasing a product. Therefore, it was hypothesized that:

H12 Perceived behavioral control (PBC) has a direct significant effect towards shopping cart abandonment (SCA).

## 3 Methodology

### 3.1 Participants

The study considered convenience sampling which garnered 1,015 valid Filipino respondents. Due to the COVID-19 pandemic, the only way to collect reliable data was through this method which is considered economical, uncomplicated, and prompt. Similar to the study of Kurata et al. (2022), the employed method would represent the general public and could be used when no additional input for the respondents would be necessary. The data was collected online through social media platforms (e.g., Facebook, Twitter, Instagram) by posting in different groups and group distribution. Hair et al. (2010), Ong et al. (2021b), and Kurata et al. (2022) explained how structural equation models with more than eight (8) variables should have more than 500 respondents to generalize the results. With that, the higher the number of respondents, the more generalizable the output would be (Hair et al., 2010). In terms of generalizability, German et al. (2022) suggested to utilize the Yamane Taro formula for representation of the general public. Given that this study was conducted in the Philippines, 62.2 Million Filipino with 95% accuracy was considered as seen in Eq. (1). German et al. (2022) indicated that 400 respondents would represent the objective of this study, which was sufficiently achieved in this study.

(1) $$n=\frac{N}{1+N\;{\left(e\right)}^{2}}$$

Collecting from December 2021–February 2022, the descriptive statistic of the demographics is presented in Table 1. Before answering the survey questionnaire, a consent form was filled out by the participants as approved by Mapua University Research Ethics Committees (Document No.: FM-RC-22-25) following Republic Act No. 10173 (Data Privacy Act of the Philippines). From the table, respondents who have knowledge and/or prior experience with online shopping as well as using online shopping applications are presented. As seen in the table below, 50.54% of the respondents were female and 49.46% of the respondents were male. The age range having the most respondents were 25–34 years old (45.9%), followed by 18–24 years old (23.4%), and 35–44 years old (21.9%). Most of the respondents were employed (79.4%) and some are students (20.3%). In terms of marital status, most of the respondents are single (59.7%) or married (39.5%). Lastly, for their monthly income/allowance, Majority have 30,001–45,000 PH P (35.9%), 15,000–30,000 PH P (32.5%), and less than 15,000 PH P (21.9%).

**Table 1:** Descriptive statistics of demographics (n = 1015).

Characteristics	Category	n	%
Gender	Male	502	49.46
	Female	513	50.54
Age	18–24 years old	238	23.40
	25–34 years old	466	45.90
	35–44 years old	222	21.90
	45–54 years old	80	7.900
	55–64 years old	8	0.800
	More than 65 years old	1	0.100
Occupation	Student	206	20.30
	Employed	806	79.40
	Unemployed	3	0.300
	Single	606	59.70
Marital Status	Married	401	39.50
	Separated	4	0.400
	Widowed	4	0.400
	Less than 15,000 PH P	222	21.90
Monthly Income/Allowance	15,000–30,000 PH P	330	32.50
	30,001–45,000 PH P	364	35.90
	45,001–60,000 PH P	74	7.300
	60,001–75,000 PH P	10	1.000
	More than 75,000 PH P	15	1.500

### 3.2 Questionnaire

The indicators for the different latent variables (*as seen in the supplementary files*) were adapted from different studies. A total of 53 constructs were considered to measure 9 latent variables. From which, the researchers employed modification towards each item to address the desired measurement for factor considered. The questionnaire considered a 5-point Likert Scale – similar to the adapted literatures to measure latent variables such as attitude (A), perceived behavioral control (PBC), and subjective norm (SN) under TPB. In addition, factors such as attribute conflict (AC), self-efficacy (SE), emotional ambivalence (EA), hesitation to check-out (HC), and choice-process satisfaction (SAT) under CAB were considered to determine factors affecting shopping cart abandonment.

### 3.3 Structural Equation Modeling

Structural Equation Modeling (SEM) is a multivariate tool utilized to determine causal relationship among latent variables. Studies relating to purchasing behavior (Prashar et al., 2017; Ong et al., 2021) and use of technology (Chuenyindee et al., 2022; Yuduang et al., 2022) have utilized SEM. These studies claimed how SEM is a powerful tool to evaluate the relationship through direct and indirect effects. Hair et al. (2010) also explored on the SEM as a multivariate tool suitable for data analysis, commonly used for human factors. However, several studies have explored on the limitations of SEM. To which, the presence of mediators and mediating effects can cause low to no significance (German et al., 2022; Fan et al., 2016; Woody, 2011). Thus, machine learning algorithms may be employed to highlight the most significant factors that promotes linear relationship despite the presence of mediators (Ong et al., 2022a; Gumasing et al., 2022). One classification technique that produces a model with high accuracy that could be utilized is the random forest classifier (RFC).

### 3.4 Random forest classifier

RFC is a classification technique that produces a higher accuracy as compared to the simple decision tree. RFC determines the best tree outcome every run, to which it creates a model that can classify significant factors affecting a dependent variable (Chen et al., 2019). Following the study of Ong et al. (2022a), correlation analysis was conducted to clean the data. Specifically, items value greater the 0.05 p-value and less than 0.20 correlation coefficient are deemed insignificant. Following which is the data aggregation technique which utilized average to specify the latent variables as input. Lastly, the min_max scalar was utilized in Python 3.8 for the data normalization process. The RFC parameters were optimized to determine the best tree. Ong et al. (2022b) suggested to determine the tree depth (4–7), training and testing ratio (60:40–90:10), criterion (gini or entropy), and splitter (best and random). A total of 6400 runs were conducted to determine the best parameter which will produce the best tree.

## 4 Results

### 4.1 Structural Equation Modeling

**Figure 4:**
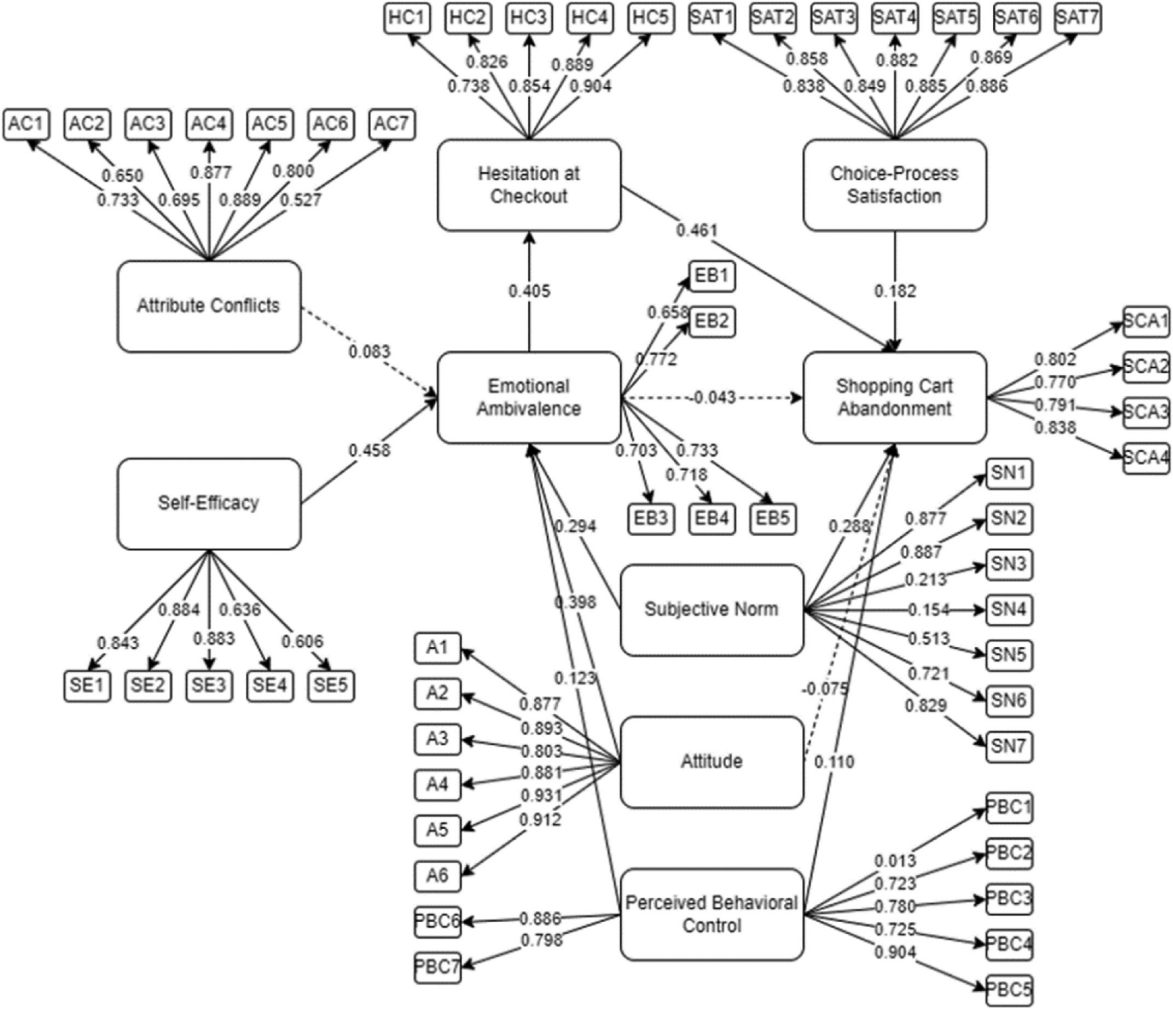
Initial SEM for online shopping cart abandonment.

The initial SEM is presented in Figure 4 ran with AMOS 25 (Ong et al., 2021). Based on the results, several indicators were not able to reach the threshold of 0.5, which were removed and deemed insignificant (Hair et al., 2010). Hair et al. (2010) suggested that removal of these indicators would enhance the model fit for the acceptability of the SEM. From which, SN3, SN4, and PBC1 were removed. The different latent variable relationship such as A on SCA, EA on SCA, and AC were removed due to insignificance (p-value greater than 0.05).

**Figure 5:**
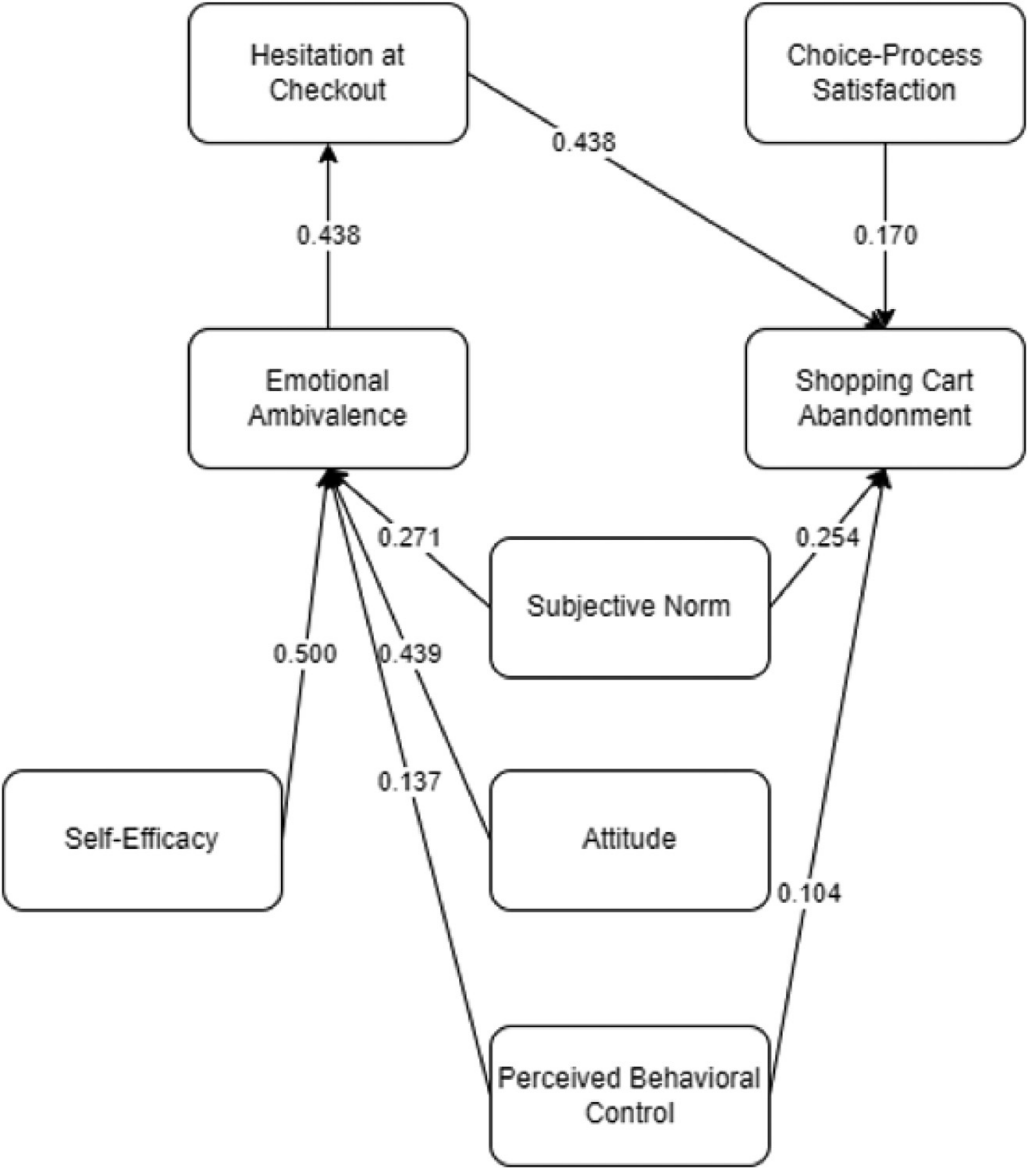
Final SEM for online shopping cart abandonment.

The final SEM for online SCA is presented in Figure 5, wherein all indicators were seen to be within the 0.50 and greater threshold. The descriptive statistics of the different constructs and factor loadings are presented in Table 2.

**Table 2:** Indicators statistical analysis.

Variable	Item	Mean	StD	Factor Loading	
				Initial	Final
Attribute Conflict	AC1	4.2700	0.80680	0.733	-
	AC2	4.1261	0.90683	0.650	-
	AC3	3.9458	0.91773	0.695	-
	AC4	4.0227	0.90281	0.877	-
	AC5	4.1212	0.87034	0.889	-
	AC6	3.8394	0.94776	0.800	-
	AC7	3.4010	0.97998	0.527	-
Attitude	A1	3.9330	0.81253	0.877	0.877
	A2	3.9281	0.81755	0.893	0.893
	A3	3.9369	0.87140	0.803	0.804
	A4	3.9557	0.85370	0.881	0.881
	A5	3.9330	0.83881	0.931	0.931
	A6	3.9931	0.81586	0.912	0.912
Self-Efficacy	SE1	3.9133	0.93811	0.843	0.843
	SE2	3.9675	0.93260	0.884	0.884
	SE3	4.0020	0.88601	0.883	0.883
	SE4	3.8463	1.00689	0.636	0.637
	SE5	3.7261	1.00387	0.606	0.607
Emotional Ambivalence	EB1	3.5596	0.92315	0.658	0.669
	EB2	3.6118	0.91398	0.772	0.782
	EB3	3.5429	0.94414	0.703	0.715
	EB4	3.5498	0.93012	0.718	0.729
	EB5	3.7714	0.86173	0.733	0.745
Hesitation at Check-out	HC1	3.6581	0.91792	0.738	0.740
	HC2	3.7271	0.93970	0.826	0.828
	HC3	3.9241	0.91296	0.854	0.855
	HC4	3.8837	0.91464	0.889	0.890
	HC5	3.8916	0.90040	0.904	0.905
Shopping Cart Abandonment	SCA1	3.5074	1.07678	0.802	0.838
	SCA2	3.4906	1.02130	0.770	0.800
	SCA3	3.3970	1.06726	0.791	0.767
	SCA4	3.4906	1.08407	0.838	0.836
Choice-process Satisfaction	SAT1	3.7951	0.80272	0.838	0.838
	SAT2	3.8601	0.80808	0.858	0.859
	SAT3	3.8000	0.84054	0.849	0.849
	SAT4	3.8404	0.83804	0.882	0.882
	SAT5	3.8502	0.82445	0.885	0.885
	SAT6	3.8946	0.82354	0.869	0.869
	SAT7	3.8473	0.82570	0.886	0.886
Subjective Norm	SN1	2.7498	1.20830	0.877	0.886
	SN2	2.6660	1.21211	0.887	0.906
	SN3	3.4049	1.07260	0.213	-
	SN4	3.5094	1.07034	0.154	-
	SN5	3.9251	0.97620	0.513	0.521
	SN6	3.1616	1.14247	0.721	0.686
	SN7	2.7754	1.28594	0.829	0.824
Perceived Behavioral Control	PBC1	2.5803	1.24638	0.013	-
	PBC2	3.8562	0.94889	0.723	0.723
	PBC3	3.8621	0.90183	0.780	0.781
	PBC4	4.1419	0.95126	0.725	0.725
	PBC5	4.1645	0.91099	0.904	0.904
	PBC6	4.0463	0.90409	0.886	0.886
	PBC7	4.3409	0.87933	0.798	0.798

**Table 3:** Model fit.

Goodness of fit measures of SEM	Parameter Estimates	Minimum cut-off	Suggested by
Incremental Fit Index (IFI)	0.881	>0.80	Gefen et al. (2000)
Tucker Lewis Index (TLI)	0.868	>0.80	Gefen et al. (2000)
Comparative Fit Index (CFI)	0.882	>0.80	Gefen et al. (2000)
Goodness of Fit Index (GFI)	0.850	>0.80	Gefen et al. (2000)
Adjusted Goodness of Fit Index (AGFI)	0.832	>0.80	Gefen et al. (2000)
Root Mean Square Error (RMSEA)	0.065	<0.07	Steiger (2007)

To determine the acceptability of the final SEM, parameters such as TLI, IFI, GFI, CFI, and AGFI were considered as seen in Table 3. As suggested by Gefen et al. (2000), these parameters should have values greater than 0.80 to have an acceptable model. Moreover, Steiger (2007) suggested to consider the Root Mean Square Error (RMSEA) with value less than 0.07. To which, all parameters considered are within the threshold and thus the final SEM is considered highly acceptable.

The use of Cronbach's alpha (CA) was used to validate the results, together with the composite reliability (CR). Ong et al. (2021) indicated that the CR and CA should have values greater than 0.70 to have a reliable set of constructs to measure the latent variable. Moreover, the Average Variance Extracted (AVE) should be greater than 0.50 to further validate the constructs utilized. The results are presented in Table 4.

**Table 4:** Composite reliability and validity.

Factor	Cronbach's α	Composite Reliability (CR)	Average Variance Extracted (AVE)
Attitude	0.954	0.955	0.781
Self-Efficacy	0.882	0.884	0.609
Emotional Ambivalence	0.898	0.850	0.531
Hesitation at Check-out	0.931	0.926	0.715
Shopping Cart Abandonment	0.892	0.885	0.657
Choice-process Satisfaction	0.955	0.955	0.752
Subjective Norm	0.844	0.881	0.605
Perceived Behavioral Control	0.827	0.917	0.650

**Table 5:** Direct, indirect, and total effects.

No	Variable	Direct Effect	P-Value	Indirect Effect	P-Value	Total Effect	P-Value
1	PBC → EA	0.137	0.010	-	-	0.137	0.010
2	A → EA	0.439	0.020	-	-	0.439	0.020
3	SN → EA	0.271	0.007	-	-	0.271	0.007
4	SE → EA	0.500	0.014	-	-	0.500	0.014
5	EA → HC	0.419	0.012	-	-	0.419	0.012
6	PBC → SCA	0.104	0.039	0.025	0.007	0.129	0.019
7	SN → SCA	0.254	0.015	0.050	0.007	0.304	0.019
8	SAT → SCA	0.170	0.009	-	-	0.170	0.009
9	HC → SCA	0.438	0.011	-	-	0.438	0.011
10	PBC → HC	-	-	0.057	0.009	0.057	0.009
11	A → HC	-	-	0.184	0.016	0.184	0.016
12	SN → HC	-	-	0.113	0.012	0.113	0.012
13	SE → HC	-	-	0.209	0.006	0.209	0.006
14	A → SCA	-	-	0.080	0.007	0.080	0.007
15	SE → SCA	-	-	0.092	0.003	0.092	0.003
16	EA → SCA	-	-	0.183	0.005	0.183	0.005

Lastly, the causal relationship of the final SEM is presented in Table 5. Testing the hypotheses, only 2 were not significant. In addition, the Common Method Bias (CMB) was tested using the Harman's Single Factor test. The result showed a 26.37% total variance, which indicates no CMB present (<50%) (Ong et al., 2021b).

### 4.2 Random forest classifier

Duarte and Pinho (2019) suggested another approach to support the findings of SEM. Chen et al. (2019) suggested utilizing RFC to discover the most significant latent variable affecting the study's objective. This is supported in the study of Fan et al. (2016) and Woody (2011) wherein they discussed how SEM produces low to no significant latent variables due to the indirect effect that may be present to measure the dependent variable. To which, analysis of variance (ANOVA) was used to examine the difference between the results of the RFC after the optimization process to highlight the best tree to determine the most significant latent variables. The summarized result from the highest obtained average accuracy is presented in Table 6.

**Table 6:** Decision tree mean accuracy (depth = 5).

Category	60:40	70:30	80:20	90:10
Random				
Gini	82.90	80.63	84.02	84.62
Std. Dev	4.236	5.387	5.650	5.626
Entropy	82.44	81.66	82.63	85.68
Std. Dev	4.541	5.096	7.443	5.920
Best				
Gini	84.00	83.00	**96.00**	88.00
Std. Dev	0.000	0.000	**0.000**	0.000
Entropy	83.52	86.51	89.00	88.00
Std. Dev	0.502	0.502	0.000	0.000

**Figure 6:**
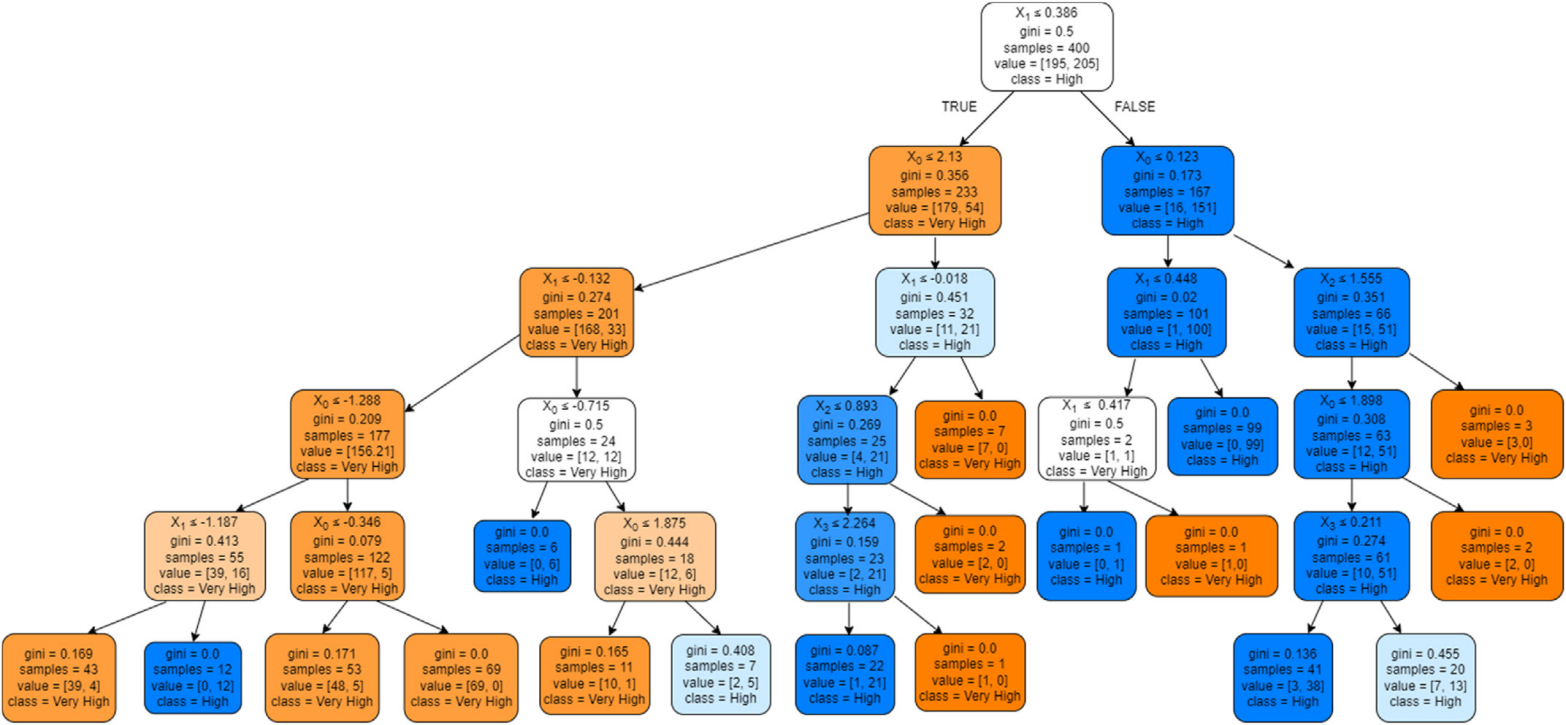
Random forest classifier model. Legends: X0 – attribute conflict (AC), X1 – attitude (A), X2 – self-efficacy (SE), X3 – emotional ambivalence (EA).

It could be deduced from the results that the parameters that would produce the best RFC model caters gini for the criterion, best as the splitter, 5 tree depth, and at 80:20 ratio. A 96.00% with 0.000 standard deviation accuracy was seen and the best RFC classification model was obtained. Presented in Figure 6 is the best RFC model to predict factors affecting online shopping cart abandonment during the covid-19 pandemic.

The parent node that would indicate SCA would be attitude (X1) that has a value of less than or equal to 0.386, which would consider attribute conflict (X0). If satisfied, it will consider attitude (X1). If the value is less than or equal to 2.13, it would consider attitude (X1) with a value of less than or equal to -0.132. To which, it would consider attribute conflict (X0) which will lead to very high SCA.

However, if the second node was not satisfied, it would consider attitude (X1) with values less than or equal to -0.018. Satisfying this would lead to self-efficacy (X2) with values less than or equal to 0.893. Then it will consider emotional ambivalence (X3) which will lead to high SCA. Not satisfying self-efficacy (X2) will lead to high SCA. If the parent node, attitude (X1) would have a value greater than 0.386, it would consider the node of attribute conflict (X0) which leads to high SCA. If attribute conflict (X0) has a value less than or equal to 0.123, it will consider attitude (X1) which leads to high SCA. Further splitting, if attitude (X1) has a value less than or equal to 0.448, it will satisfy attitude (X1) which will lead to a very high SCA. Lastly, if the value of attitude (X1) is less than or equal to 0.417, it will result to high SCA.

## 5 Discussion

The growth of e-commerce was seen to heightened during the COVID-19 pandemic. However, the rise of shopping cart abandonment also followed leading to the loss in sales among online retailers. This study integrated the cognition-affective-behavior paradigm with TPB to assess and predict factors affecting online shopping cart abandonment. With the utilization of SEM and RFC, several findings were highlighted. The RFC result presented that AC, SE, EA, and A were significant latent that would lead to high or very high SCA. AC was considered to be insignificant for the SEM result. However, the RFC indicated that this factor is significant to SCA which will affect A, SE, EA leading to very high SCA.

AC may cause cognitive dissonance, a psychiatric disorder that can cause contradictory feelings. Consumers with a wide range of happy and negative emotions are more likely to be ambivalent. SE is one of the antecedents of emotional conflicts. The resulting emotional conflicts lead to HC and ultimately to SCA. EA is a significant indicator of remarkably high SCA. Based on the survey, EA is positive, favorable, wise, beneficial, pleasant, and good. This reveals that people have a pleasing experience that motivates them to use mobile shopping applications, thus making it significant. Lastly, A are defined as the degree to which an individual's feelings toward a psychological object are favorable or unfavorable (Ajzen and Fishbein, 2000). More favorable A are perceived as forecasting higher intentions to behave, indicating that A is a strong indicator on intention. Yang et al. (2007) also presented and supports the result of this study. On the other hand, Chea and Luo (2008) indicated that perception among consumers are seen with AC. This contrast the positive findings in this study since the consumers find positive attributes when it comes to shopping online. If, however, consumers would not find online shopping to be interesting, entertaining, convenient, and safe, a negative implication would be the result.

The results indicated that SE had the highest significant effect on EA (β: 0.500; p = 0.014). This is consistent with the result of RFC, wherein SE and EA were significant indicators that will lead to very high and high SCA, respectively. From the indicators, it was seen that people are confident, proficient to shop online given enough time, and that they have the means to ask for help in using the online shopping application. Mobile shopping applications have been seen to be quite challenging to utilize based on the indicators, in a way that people need guidance or help in using it. It could be deduced that a huge factor comes from the difficulty to navigate the system. SE influenced one's own emotion, which indicated that it can elicit negative feelings like the attractiveness to mobile shopping and the benefits of it. On the other hand, low SE leads to high EA and individuals would not engage to it. Fullagar et al. (2013) indicated that SE greatly affects one's decision. If a person has a low SE, he/she is prone to have a higher EA making that person possibly abandon his/her online shopping cart. In contrast, the study of Ong et al. (2021) indicated that shopping online would be deemed least likely chosen by people since consumers would highlight more the physical appearance and feel of the item before purchasing. However, the findings of this study indicated that consumers are more focused on completion of task and ease of use that is why they are more likely to choose and use the online shopping. In that case, if complications arise while the technology is being used, then negative aspects toward an individual SE would be evident.

Second, HC was seen to have a direct significant effect on SCA (β: 0.438; p = 0.011). It was evident that people hesitated, thought twice, and waited a while on completing the checkout stage while using a mobile device. Furthermore, people consume time deciding and clicking the final payment button to purchase. This reveals the behavior of people faced with a decision dilemma during the check-out process mainly because of some antecedents. This is in line with the study of Sondhi et al. (2017) which stated that behavior is an outcome of the cognitive dissonance concerning an online purchase decision. The result of the study showed that there is more likelihood of SCA when the incidence of perceived risk is higher. Moreover, Huang et al. (2018) confirms the results of the study, wherein HC partially mediates the effect of EA on SCA. In contrast, the behavioral aspects of a person was seen to affect the consumer's HC rather than the emotional aspect.

Third, A was seen to have a direct significant effect on EA (β: 0.439; p = 0.020). Similar to the result from RFC, A is a significant indicator that will lead to very high SCA. Based on the indicators, the A of people toward EA is positive, favorable, wise, beneficial, pleasant, and good. This reveals that people have a pleasing experience that motivates them to use mobile shopping applications. This result is similar to that of Huang et al. (2018). It was explained that interpersonal conflicts (such as between attitudes and reference groups) were positively related to EA. Cecchini (2021) mentioned how the affective, cognitive, and behavioral components under A affects EA. Van Harreveld et al. (2009) also indicated the same analogy.

Fourth, EA was seen to have a significant effect on HC (β: 0.419; p = 0.012). The result showed that people have a high hesitation during checkout, which has something to do with the EA. Consumers tend to think twice and critically to make sure that they will check out the items that are in their cart. Shoppers often use their mobile phones to shop online to check the prices or browse items. With the EA being a significant factor, it makes them more cautious with the risk that can occur upon purchasing, thus abandoning their shopping cart. Mobile shopping has been rapidly becoming the most popular medium of online shopping these days. However, SCA also increased the rate of the desktop. It states that mobile shopping is easier, yet EA comes into play where a person having low EA would lead to abandonment of shopping cart. In contrast, Ong et al. (2021) indicated that people are sure upon purchasing of items. In the results, it was seen that more people, especially during the COVID-19 pandemic would be indecisive which leads to online SCA. It could be deduced that the economic aspect – down during the COVID-19 pandemic, affected the cognitive aspect of consumers as to why there are contrasting results.

In terms of SN, it has a direct significant effect on EA (β: 0.271; p = 0.007) and SCA (β: 0.254; p = 0.015). From the indicators, it can be seen that people feel the need to shop online, are under the social pressure, and expectations of doing so from the people around them. With this, consumers consider their decision on other people if they will purchase or not. In terms of SN, Vijaysarathy and Jones (2000) stated that communicating through visual, text, and sound can also positively or negatively affect consumers' purchase intention to continue shopping. In addition, they can be influenced by their family, peers, and even the media since people often act based on others' perception. Liu et al. (2011) found that SN has a positive effect on purchase intention. If the consumer feels happy and satisfied with their shopping experience, there is an intention to purchase. The social pressure rooted from the SN can have an effect on the person's EA. Likewise, Chea and Luo (2008), van Harreveld et al. (2009), and Chang (2011) supports this finding. When there is a clear social norm from people, an ambivalent attitude can be present (Pillaud et al., 2018). This implies that SN has an effect on EA and SCA.

The results also showed that PBC directly affects EA (β: 0.137; p = 0.010) and SCA (β: 0.104; p = 0.039). It could be deduced that people who are utilizing online shopping are not indecisive, conflicted, or even uncertain when purchasing. They would know what to do upon the utility of the application. Additionally, it can also be seen that people often set items in the shopping cart and either close the webpage immediately or leave the order. In addition, it can also be said that people often leave or abandon their items in their carts, leading to SCA. Several researchers have claimed that several factors influence SCA. Buying intent, price promotion, experimental purpose, organizational intent, and research and information search are all factors that influence online purchasing behavior (Kukar-Kinney and Close, 2010). According to George (2004) and Hsu et al. (2011), trust is a PBC determining SCA behavior. Additionally, previous research on risk behaviors has demonstrated that taking into account TPB characteristics and previous emotions enhances predictions about the likelihood of repeating them (Caballero et al., 2007). This implies that PBC affects both EA and SCA.

The findings revealed that SAT has a minor direct impact on SCA (β: 0.170; p = 0.009). According to the indicators, people are contented, happy to shop online when given the opportunity, and can choose from a variety of products using an online shopping application. The process of picking which items to add to the cart piques the users' interest so that people are willing to choose from a variety of options. The selection of items available are also significant. Availability for choices, quality, and assortment are measurement of satisfaction under SAT (Fitzsimons, 2000). The satisfaction of consumers with the process in decision making rises when options and information are screened. Consumers would consider it challenging to consider and determine the proper strategy with lack of product category knowledge, especially with numerous product options (Heitmann et al., 2007). Thus, this indicates that SCA is significantly influenced by SAT. However, studies such as that of Lee and Workman (2015) indicated that consumers are affected by their brand loyalty attitude. This, however, is not prevalent in this study since online shopping provides more opportunity for convenience when it comes to choosing an item. In addition, it was seen that consumers want to take their time in considering different available options for them. Thus, contradicts the implications from Lee and Workman (2015).

Overall, indicators under SCA included the frequency of placing items in the cart but not necessarily buying in the same instance, the frequency of logging off the mobile application, and the frequency of leaving items in the cart but not buying them at all. In addition, the factors that have the most significant effect which led to high or very high SCA are AC, A, SE, and EA. Thus, ease of use, convenience, and safety of the mobile online shopping applications should be considered in order to lessen online SCA.

### 5.1 Theoretical contributions

The framework of the CAB paradigm with the extension of TPB measures the relationship between factors that leads to the online SCA of customers holistically. CAB paradigm serves as a foundation for describing and explaining consumer behavior. Specifically, this paradigm covers the cognitive and emotional aspect of a person. Based from the findings of the study, it could be deduced that self-efficacy played the most contributing factor it terms of cognitive aspects while emotional ambivalence on the emotional aspects. These two main factors, antecedents or preceded by choice-process satisfaction and attribute conflicts would lead to prevalent effects for a consumer's positive or negative decision towards intention or action. From the study, it could be deduced that the CAB paradigm measures consumer's behavior emotional and cognitive aspects which is deemed important for businesses in decision making. However, the behavioral aspects which would help business endeavors are need as to why the study evaluated the domains under TPB as an integration to the model.

TPB represents customers’ actual control over their behavior. With the integration of this, all consumer behavioral aspects are covered and could indicate holistic measurement of online SCA. The integrated framework may also be utilized and extended to measure behavioral aspects of online shopping in other platforms, purchasing behavior, and consumer intention. In addition, the results contribute by giving the businesses/retailers a clear overview and deeper understanding of the behavioral pattern of consumers in the online shopping industry. With this as a basis, retailers will be able to formulate business strategies covering all aspects of consumers when it comes to emotional, cognitive, and behavioral aspects that will lessen the online SCA, improve customer experience, and drive more profit.

### 5.2 Practical and managerial contributions

Businesses and retailers may take advantage of the factors identified in this study to determine instances and frequency of online SCA. From the results, businesses and retailers may highlight the aspects of AC, A, SE, and EA to reduce SCA. From the findings, EA indicators provided pleasing experiences that motivate consumers to use mobile shopping applications. Example, when applications would tend to lag or items would take long to load (*e.g.* pictures) – people would likely consider their phone to be malfunctioning or directly have negative feelings for continuance of purchase since the application is not working well. In line with this, businesses should consider an investment in servers to provide prompt utility with the mobile shopping experience. Second, pleasing platform when it comes to navigation should be considered to appeal among different generation. This would provide ease of use and convenience among users which would tap their EA.

Under AC, indicators provided aspects of the convenience, entertaining, timely, and safe as perception to platforms during online shopping. Example of which is how Shoppee as an online shopping platform provides users with games to gain points, promotions through coupons won in games, and discounts from the coins they earned from games. This would also provide users with a control over what they would do (*i.e.* play games to gain promotions or directly shop). E-commerce managers and promoters may capitalize on this attribute by providing entertainment, promotions, and discounts among consumers to have great implication upon application utility. Thus, it can cover the positive attitude consumers would have upon application usage.

SE was seen to have an effect towards online SCA. The indicators provided insights on how consumers would utilize the application in a way that it appeals to the general public – focusing mainly on the navigation, built-in help facility, and proficient shopping. Similar to the Quality Pet online shop, help desk are available when consumers are in need. Navigation has been seen to be efficient with proper categorization and tab linkage, as well as notification through single click or emails are evident. This provides confidence among consumers, proficient application navigation, empathy, and time.

With that, SCA can be reduced through managing product selection and presentation, as well as promotional activities. By analyzing and knowing the behavioral, cognitive, and emotional aspects among consumers, retailers or e-commerce may effectively tailor personalize and efficient experiences among their users. Moreover, the framework and results of this study may be considered for application and extension to create business strategies, determine factors affecting buying behavior, and even develop business models for different retail and wholesale industries worldwide.

One solution that can also be implemented in order to lessen or refrain the consumer from abandoning their shopping cart is to max out the available stocks in order to accommodate a large volume of consumer who want to check-out the item. In addition, provisions on shopping cart storage may be limited to certain days as suggestion to have inventories for consumers to purchase. It can also be suggested that shops can promote vouchers and package deals in order to attract more customers as well. Prasetyo et al. (2020) also suggested promotion of items through online advertisement since it was seen that it affects customers intention. Retailers and e-commerce should capitalize on these findings since people are more reliant on using applications since the COVID-19 pandemic and lockdown implementations. Thus, consumers would want to buy items and necessities online to feel safe, avoid getting sick, and avoid COVID-19 contraction. Since other countries are still closed due to the COVID-19 spread, the findings of this study is still timely and relevant.

For users, benefit when it comes to the convenience, ease of use, provided platform for access to items in the comfort of their home with less hassle would be evident. The way e-commerce and retailers would achieve this is by considering what factors affects the online SCA. As stated, several strategies may be developed based from the example to have a more entertaining, empathetic, user-friendly, and more options among users which would benefit customers and businesses to gain what they need. For example, it was suggested by Gumasing et al. (2022) how online grocery would provide ease of use, timely, and relevant day-to-day needs among users when the necessities and items needed are a single click. Their study justified that convenience among users should be the most important factor platforms should consider so consumers would rely on using the application when it comes to their tangible needs.

### 5.3 Limitations

Despite the significant findings and holistic approach in methodology, several limitations were still evident. First, this study considered SEM and RFC. Other machine learning algorithms may be utilized such as deep learning neural networks, K-Nearest Neighbors, and Naive Bayes to classify and cluster indicators and demographic factors, which may help in building a business model and strategy among online retail markets. In addition, interviews may be conducted to highlight and determine other factors which may affect online SCA that were not included in this study. A qualitative-quantitative study may be conducted to assess and create portfolio for marketing strategy among the results that may be obtained. Lastly, the consideration of focused group demographics may be highlighted to segment customers according to their age group, needs, and wants. This may help businesses to develop a more realistic and generalized business model.

## 6 Conclusion

Online shopping has accelerated due to the pandemic. To which, one of the key factors that is affecting the e-commerce industry is the occurrence of online SCA. This study aimed to predict factors that affect online SCA among Filipinos during the COVID-19 pandemic. The extension of the Cognitive-Affective Behavior paradigm (CAB) with the Theory of Planned Behavior (TPB) was utilized to cover all the consumers’ behavioral aspects. The assessment utilized a SEM-RFC hybrid to classify factors affecting SCA. From a total of 1,015 people who voluntarily participated in the study and responded to an online survey, it was evident that SEM-RFC hybrid provided consistent results. Nine latent were measured namely: (1) attribute conflicts, (2) attitude, (3) self-efficacy, (4) emotional ambivalence, (5) hesitation at check-out, (6) mobile shopping cart abandonment, (7) choice-process satisfaction, (8) subjective norms, and (9) perceived behavioral control. Through the integration, it was seen that holistic measurement among consumers when it comes to behavioral, cognitive, and emotional aspects were covered.

Results of SEM showed that PBC, A, SN and SE affected EA. In line with this, EA directly affects HC since mixed feelings can make a consumer hesitant to buy or purchase a certain product, and this hesitation will lead to SCA. From the inclusion of TPB in the CAB paradigm, SN and PBC were shown to directly affect SCA. SN shows how consumers perceive that people have the capacity to abandon their online shopping carts, which in turn, the consumer has the tendency to do the same thing. In terms of PBC, the consumer has the confidence that they are capable of SCA. As consumers felt the limited options and availability of products of online shops during the early surge of COVID-19, this shows how they were mostly unsatisfied and displeased with their shopping experience which led to them discontinuing their purchases. For RFC, results showed that AC, A, SE and EA were the only significant latent variables which led to high or very high SCA. Amidst the pandemic, consumers still value the ease of use, convenience, and safety of the mobile online shopping applications that they have, which they do not positively experience at this time. These can be considered when forming and adjusting marketing strategies for retailers in order to prevent customer loss. The findings and framework utilized in this study may be applied and extended to other participants and businesses worldwide.

## Declarations

### Author contribution statement

Ardvin Kester S. Ong; Marjorie Joy R. Dejucos; Mary Anne F. Rivera; John Vincent D.J. Muñoz; Miguel S. Obed: Conceived and designed the experiments; Performed the experiments; Analyzed and interpreted the data; Contributed reagents, materials, analysis tools or data; Wrote the paper.

Kirstien Paola E. Robas: Analyzed and interpreted the data; Contributed reagents, materials, analysis tools or data; Wrote the paper.

### Funding statement

This work was supported by Mapúa University Directed Research for Innovation and Value Enhancement (DRIVE).

### Data availability statement

Data will be made available on request.

### Declaration of interest's statement

The authors declare no conflict of interest.

### Additional information

No additional information is available for this paper.

## Appendix A Supplementary data

Questionnaire.docx Questionnaire.docx

## References

[bib1] Ajzen I., Fishbein M. (2000). Attitudes and the attitude-behavior relation: reasoned and automatic processes. Eur. Rev. Soc. Psychol..

[bib3] Caballero A., Carrera P., Muñoz D., Sánchez F. (2007). Emotional ambivalence in risk behaviors: the case of occasional excessive use of alcohol. Spanish J. Psychol..

[bib4] Cecchini D. (2021). Experiencing the conflict: the rationality of ambivalence. J. Value Inq..

[bib5] Chang C. (2011). Feeling ambivalent about going green. J. Advert..

[bib7] Chea S., Luo M. (2008). Post-adoption behaviors of E-service customers: the interplay of cognition and emotion. Int. J. Electron. Commer..

[bib8] Chen J., Li Q., Wang H., Deng M. (2019). A machine learning ensemble approach based on random forest and radial basis function neural network for risk evaluation of regional flood disaster: a case study of the yangtze river delta, China. Int. J. Environ. Res. Publ. Health.

[bib9] Cho J. (2003). Likelihood to abort an online transaction: influences from cognitive evaluations, attitudes, and behavioral variables. Inf. Manag..

[bib10] Cho C.H., Kang J., Cheon H.J. (2006). Online shopping hesitation. Cyberpsychol. Behav..

[bib11] Chuenyindee T., Ong A.K.S., Prasetyo Y.T., Persada S.F., Nadlifatin R., Sittiwatethanasiri T. (2022). Factors affecting the perceived usability of the COVID-19 contact-tracing application “Thai chana” during the early COVID-19 omicron period. Int. J. Environ. Res. Publ. Health.

[bib12] Conner M., Povey R., Sparks P., James R., Shepherd R. (2003). Moderating role of attitudinal ambivalence within the theory of planned behaviour. Br. J. Soc. Psychol..

[bib13] Data Reportal (2021). https://datareportal.com/reports/digital-2021-philippines.

[bib16] Duarte P., Pinho J.C. (2019). A mixed methods UTAUT2-based approach to assess mobile health adoption. J. Bus. Res..

[bib17] Egeln L., Joseph J. (2012). Shopping cart abandonment in online shopping. Atlantic Marketing Journal.

[bib18] Ekta Mehta M., Sehgal B. (2020). Buying practices of homemakers through cashless transaction. Advances in Research.

[bib19] Fan Y., Chen J., Shirkey G., John R., Wu S.R., Park H., Shao C. (2016). Applications of structural equation modeling (SEM) in ecological studies: an updated review. Ecological Processes.

[bib20] Fitzsimons G.J. (2000). Consumer response to stockouts. J. Consum. Res..

[bib21] Fox M. (2020). https://www.dynamicyield.com/blog/shopping-cart-abandonment-ebook-announcement/.

[bib22] Fullagar C., Knight P., Sovern H. (2013). Challenge/skill balance, flow, and performance anxiety. Appl. Psychol..

[bib23] Gefen D., Straub D., Boudreau M.C. (2000). Structural equation modeling and regression: guidelines for research practice. Commun. Assoc. Inf. Syst..

[bib24] George J. (2004). The theory of planned behavior and Internet purchasing. Internet Res..

[bib25] German J.D., Redi A.A.N.P., Prasetyo Y.T., Persada S.F., Ong A.K.S., Young M.N., Nadlifatin R. (2022). Choosing a package carrier during COVID-19 pandemic: an integration of pro-environmental planned behavior (PEPB) theory and service quality (SERVQUAL). J. Clean. Prod..

[bib27] Gumasing Ma.J.J., Prasetyo Y.T., Persada S.F., Ong A.K.S., Young M.N., Nadlifatin R., Redi A.A.N.P. (2022). Using online grocery applications during the COVID-19 pandemic: their relationship with open innovation. J. Open Innov.Technol. Market, and Compl..

[bib28] Hair Joseph, Black William, Babin Barry, Anderson Rolph (2010).

[bib29] Haque A., Khatibi A. (2005). E-Shopping: current practices and future opportunities towards Malaysian customer perspective. J. Soc. Sci..

[bib31] Heitmann M., Lehmann D.R., Herrmann A. (2007). Choice goal attainment and decision and consumption satisfaction. J. Market. Res..

[bib32] Ho A.L. (2011). https://business.inquirer.net/22101/multiply-social-shopping-site-is-here-to-stay.

[bib33] Hsu H., Ye C., Chi C., Chan C. (2006). A longitudinal investigation of continued online shopping behavior: an extension of the theory of planned behavior. Int. J. Hum. Comput. Stud..

[bib34] Hsu C., Chang K., Chen M. (2011). Flow experience and internet shopping behavior: investigating the moderating effect of consumer characteristics. Syst. Res. Behav. Sci..

[bib35] Huang G.-H., Korfiatis N., Chang C.-T. (2018). Mobile shopping cart abandonment: the roles of conflicts, ambivalence, and hesitation. J. Bus. Res..

[bib37] Jiang D., Zhang G., Wang L. (2021). Empty the shopping cart? the effect of shopping cart item sorting on online shopping cart abandonment behavior. Journal of Theoretical and Applied Electronic Commerce Research.

[bib39] Katrina B., Benedict L. (2021). Who are the Philippines’ online shoppers?. Janio.

[bib40] Khatibi A., Haque A. (2006). E-Commerce: a study on internet shopping in Malaysia. J. Appl. Sci..

[bib41] Kukar-Kinney M., Close A.G. (2010). The determinants of consumers’ online shopping cart abandonment. J. Acad. Market. Sci..

[bib42] Kumar A., Kashyap A. (2018). Leveraging utilitarian perspective of online shopping to motivate online shoppers. Int. J. Retail Distrib. Manag..

[bib43] Kurata Y.B., Prasetyo Y.T., Ong A.K.S., Nadlifatin R., Chuenyindee T. (2022). Factors affecting perceived effectiveness of Typhoon Vamco (Ulysses) flood disaster response among Filipinos in Luzon, Philippines: an integration of protection motivation theory and extended theory of planned behavior. Int. J. Disaster Risk Reduc..

[bib44] Lee A.Y., Aaker J.L. (2004). Bringing the frame into focus: the influence of regulatory fit on processing fluency and persuasion. J. Pers. Soc. Psychol..

[bib45] Lee B.K., Lee W.N. (2004). The effect of information overload on consumer choice quality in an on-line environment. Psychol. Market..

[bib46] Lee S.-H., Workman J.E. (2015). Compulsive buying and branding phenomena. Journal of Open Innovation: Technology, Market, and Complexity.

[bib47] Liu C., Forsythe S., Black W. (2011). Beyond adoption: sustaining online shopping. Int. Rev. Retail Distrib. Consum. Res..

[bib48] Magkilat B.C. (2020). https://mb.com.ph/2020/10/14/ph-posts-highest-growth-in-shopping-apps-usage-in-asean-2/.

[bib49] Mishra S., Malhotra G., Tiwari S.R. (2021). Moderating effect of cognitive conflict on the relationship between value consciousness and online shopping cart abandonment. Int. Rev. Retail Distrib. Consum. Res..

[bib50] Moore S., Mathews S. (2006). An exploration of online shopping cart abandonment syndrome – a matter of risk and reputation. J. Website Promot..

[bib51] Nonato V. (2020). https://www.onenews.ph/articles/add-to-cart-filipino-online-shopping-grows-by-57-percent-highest-in-southeast-asia-e-commerce-group-finds%20of%20Personality%20and%20Social%20Psychology.

[bib52] Ong A.K.S., Cleofas M.A., Prasetyo Y.T., Chuenyindee T., Young M.N., Diaz J.F.T., Nadlifatin R., Redi A.A.N.P. (2021). Consumer behavior in clothing industry and its relationship with open innovation dynamics during the COVID-19 pandemic. J. Open Innov. Technol. Mark. Complex.

[bib53] Ong A.K.S., Prasetyo Y.T., Lagura F.C., Ramos R.N., Sigua K.M., Villas J.A., Young M.N., Diaz J.F.T., Persada S.F., Redi A.A.N.P. (2021). Factors affecting intention to prepare for mitigation of “the big one” earthquake in the Philippines: integrating protection motivation theory and extended theory of planned behavior. Int. J. Disaster Risk Reduc..

[bib54] Ong A.K.S., Prasetyo Y.T., Velasco K.E.C., Abad E.D.R., Buencille A.L.B., Estorninos E.M., Cahigas M.M.L., Chuenyindee T., Persada S.F., Nadlifatin R., Sittiwatethanasiri T. (2022). Utilization of random forest classifier and artificial neural network for predicting the acceptance of reopening decommissioned nuclear power plant. Ann. Nucl. Energy.

[bib55] Ong A.K.S., Chuenyindee T., Prasetyo Y.T., Nadlifatin R., Persada S.F., Gumasing Ma.J.J., German J.D., Robas K.P.E., Young M.N., Sittiwatethanasiri T. (2022). Utilization of random forest and deep learning neural network for predicting factors affecting perceived usability of a COVID-19 contact tracing mobile application in Thailand “ThaiChana. Int. J. Environ. Res. Publ. Health.

[bib56] Periabras R.C. (2012). Multiply.com launches enhanced e-commerce platform. The Manila Times.

[bib57] Peterson S. (2021). https://www.namogoo.com/blog/conversion-rate-optimization/shopping-cart-abandonment-effects/.

[bib58] Pillaud V., Cavazza N., Butera F. (2018). The social utility of ambivalence: being ambivalent on controversial issues is recognized as competence. Frontiers.

[bib60] Prasetyo Y.T., Castillo A.M., Salonga L.J., Sia J.A., Seneta J.A. (2020). Factors affecting perceived effectiveness of COVID-19 prevention measures among Filipinos during enhanced community quarantine in luzon, Philippines: integrating protection motivation theory and extended theory of planned behavior. Int. J. Infect. Dis..

[bib61] Prashar S., Sai Vijay T., Parsad C. (2017). Effects of online shopping values and website cues on purchase behaviour: a study using S–O–R framework. Vikalpa. The Journal for Decision Makers.

[bib62] Priester J.R., Crano W., Burgoon M. (2002). Mass Media and Drug Prevention: Classic and Contemporary Theories and Research.

[bib64] Reyes T. (2016). INFOGRAPHIC: fast facts on online shopping in PH. Rappler.

[bib65] Rubin D., Martins C., Ilyuk V., Hildebrand D. (2020). Online shopping cart abandonment: a consumer mindset perspective. J. Consum. Market..

[bib66] Russell C.A., Russell D.W., Klein J. (2011). Ambivalence toward a country and consumers’ willingness to buy emblematic brands: the differential predictive validity of objective and subjective ambivalence measures on behavior. Market. Lett..

[bib67] Serrano S. (2022). https://www.barilliance.com/cart-abandonment-rate-statistics/.

[bib68] Sondhi N. (2017). Segmenting & profiling the deflecting customer: understanding shopping cart abandonment. Procedia Comput. Sci..

[bib69] Song J.-D. (2019). A study on online shopping cart abandonment: a product category perspective. J. Internet Commer..

[bib71] Statista (2021). https://www.statista.com/statistics/1125455/e-commerce-market-size-philippines/.

[bib72] Statista (2021). https://www.statista.com/statistics/457078/category-cart-abandonment-rate-worldwide/.

[bib73] Statista (2021). https://www.statista.com/statistics/1125430/philippines-e-commerce-activities-internet-users/.

[bib74] Statista (2021). https://www.statista.com/outlook/dmo/ecommerce/philippines.

[bib76] Steiger J.H. (2007). Understanding the limitations of global fit assessment in structural equation modeling. Pers. Indiv. Differ..

[bib77] Suki N.M., Ramayah T., Suki N.M. (2008). Internet shopping acceptance: examining the influence of intrinsic versus extrinsic motivations. Direct Mark. Int. J..

[bib78] Suls J., Wheeler L. (2000). Handbook of Social Comparison.

[bib79] Toth S. (2019). https://blog.recart.com/9-frightening-cart-abandonment-facts-can-learn/.

[bib80] van Harreveld F., van der Pligt J., de Liver Y.N. (2009). The agony of ambivalence and ways to resolve it: introducing the MAID model. Pers. Soc. Psychol. Rev..

[bib82] Verhoef P.C., Langerak F. (2001). Possible determinants of consumers’ adoption of electronic grocery shopping in The Netherlands. J. Retailing Consum. Serv..

[bib83] Vijaysarathy L., Jones J.M. (2000). Intentions to shop using internet catalogues: exploring the effects of product types, shopping orientations, and attitudes towards computers. Electron. Mark..

[bib84] Wang S., Cheah J.-H., Lim X.-J., Leong Y.C., Choo W.C. (2022). Thanks Covid-19, I'll reconsider my purchase: can fear appeal reduce online shopping cart abandonment?. J. Retailing Consum. Serv..

[bib86] Woody E. (2011). An SEM perspective on evaluating mediation: what every clinical researcher needs to know. J. Exp. Psychol..

[bib87] Wu F. (2003). The (post-) socialist entrepreneurial city as a state project: shanghai's reglobalisation in question. Urban Stud..

[bib88] Yang B., Lester D., James S. (2007). Attitudes toward buying online as predictor of shopping online for British and American respondents. Cyberpsychol. Behav.: the impact of the Internet, multimedia and virtual reality on behavior and society.

[bib89] Yuduang N., Ong A.K.S., Prasetyo Y.T., Chuenyindee T., Kusonwattana P., Limpasart W., Sittiwatethanasiri T., Gumasing Ma.J.J., German J.D., Nadlifatin R. (2022). Factors influencing the perceived effectiveness of COVID-19 risk assessment mobile application “MorChana” in Thailand: UTAUT2 approach. Int. J. Environ. Res. Publ. Health.

