# Supplementary material for: Utilizing SEM-RFC to predict factors affecting online shopping cart abandonment during the COVID-19 pandemic
Source: Heliyon. 2022 Oct 28;8(11):e11293. doi: 10.1016/j.heliyon.2022.e11293 (PMC9638758; doi:10.1016/j.heliyon.2022.e11293)
Supplement: Questionnaire.docx [file mmc1.docx]

Items with references

| **Factors** | **Items** | **Constructs** | **References** |
| --- | --- | --- | --- |
| Attributes conflicts (AC) | AC1 | I think mobile shopping is convenient. | Huang et al. (2018) |
|  | AC2 | I think mobile shopping is timely. |  |
|  | AC3 | I think mobile shopping is fast. |  |
|  | AC4 | I think mobile shopping is entertaining. |  |
|  | AC5 | I think mobile shopping is interesting. |  |
|  | AC6 | I think mobile shopping is fashionable. |  |
|  | AC7 | I think mobile shopping is safe. |  |
| Attitudes (A) | A1 | My attitude toward mobile shopping is positive. | Huang et al. (2018) |
|  | A2 | My attitude toward mobile shopping is favorable. |  |
|  | A3 | My attitude toward mobile shopping is wise. |  |
|  | A4 | My attitude toward mobile shopping is beneficial. |  |
|  | A5 | My attitude toward mobile shopping is pleasant. |  |
|  | A6 | My attitude toward mobile shopping is good. |  |
| Self-efficacy (SE) | SE1 | I am proficient in using mobile devices for shopping. | Davis and Tuttle (2013);  Venkatesh et al. (2003) |
|  | SE2 | I feel confident that I can use mobile devices for shopping. |  |
|  | SE3 | I could shop using mobile devices if I had enough time to complete the task. |  |
|  | SE4 | I could shop using mobile devices if someone showed me how to do it first. |  |
|  | SE5 | I could shop using mobile devices if I had only the built-in help facility for assistance. |  |
| Emotional ambivalence (EA) | EA1 | I have strong emotions for mobile shopping. | Chang (2011); Priester et al. (2007) |
|  | EA2 | I do not feel conflicted when thinking about mobile shopping. |  |
|  | EA3 | I am not indecisive about mobile shopping. |  |
|  | EA4 | I am not uncertain about mobile shopping. |  |
|  | EA5 | I can make up my mind one way or another about what is the best course of action for me to take regarding mobile shopping. |  |
| Hesitation at check-out (HC) | HC1 | I have hesitations to complete the checkout stage for selected items while shopping using my mobile device. | Cho et al. (2006); Wong and Yeh (2009) |
|  | HC2 | It has taken some time for me to click the final payment button to purchase products via a  mobile device. |  |
|  | HC3 | I have thought twice at the checkout stage for a purchase via a mobile device. |  |
|  | HC4 | I have spent some time deciding whether to press the payment button in a mobile shopping task. |  |
|  | HC5 | I have waited a while thinking about whether to finish the checkout process for items in the  final payment stage. |  |
| Mobile shopping cart abandonment (SCA) | SCA1 | How often do you place an item in the shopping cart, but do not buy it during the same session? | Kukar-Kinney and Close (2010) |
|  | SCA2 | How often do you close the webpage, or log off the mobile shopping application before you buy the item(s) in your shopping cart? |  |
|  | SCA3 | How often do you abandon your mobile shopping cart? |  |
|  | SCA4 | How often do you leave items in your mobile shopping cart without buying them? |  |
| Choice-process satisfaction (SAT) | SAT1 | How satisfied were you with your experience of deciding which products option to choose? | Fitzsimons (2000);  Griffin and Brioniarczyk (2010) |
|  | SAT2 | How happy were you with the process of choosing items you intended to buy? |  |
|  | SAT3 | I would be happy to choose from the same set of product options on my next purchase. |  |
|  | SAT4 | I found the process of deciding which products to put in the shopping cart interesting. |  |
|  | SAT5 | I thought the selection of products was good. |  |
|  | SAT6 | Several good options were available for me to choose between. |  |
|  | SAT7 | I found the process of deciding which product(s) to put in the mobile shopping cart  encouraging/pleasant. |  |
| Subjective Norms (SN) | SN1 | I feel obligated to shop online. | Ong et al. (2021) |
|  | SN2 | I feel that I am under social pressure to shop online. | Ataei et al. (2021) |
|  | SN3 | My family supports me when purchasing online. | Ong et al. (2021) |
|  | SN4 | My close friends support me when purchasing online. |  |
|  | SN5 | Most of the people I know are shopping online. | Song and Shi (2020) |
|  | SN6 | Most of the people who surround me expect me to shop online. | Kahlor et al. (2019) |
|  | SN7 | If people around me shop online, I feel that I need to do the same thing. | Du and Pan (2021) |
| Perceived Behavioral Control (PBC) | PBC1 | My online shopping habit is becoming alarming. | Ong et al. (2021a) |
|  | PBC2 | I have the capability to shop online. | Song and Shi (2020) |
|  | PBC3 | I feel that it is easy to utilize the online shopping application. | Prasetyo et al. (2020) |
|  | PBC4 | I have the confidence that I can control myself when shopping online. | Heidenreich et al. (2020) |
|  | PBC5 | I know how to search for products that I need to shop online. | Kahlor et al. (2019) |
|  | PBC6 | I can easily and readily access information about the online shop application. |  |
|  | PBC7 | I believe that deciding whether to shop online is up to me. | Ong et al. (2021) |
